# Editorial: Molecular Network Study of Pituitary Adenomas

**DOI:** 10.3389/fendo.2020.00026

**Published:** 2020-02-18

**Authors:** Xianquan Zhan, Dominic M. Desiderio

**Affiliations:** ^1^University Creative Research Initiatives Center, Shandong First Medical University, Shandong, China; ^2^Key Laboratory of Cancer Proteomics of Chinese Ministry of Health, Xiangya Hospital, Central South University, Changsha, China; ^3^State Local Joint Engineering Laboratory for Anticancer Drugs, Xiangya Hospital, Central South University, Changsha, China; ^4^Department of Oncology, Xiangya Hospital, Central South University, Changsha, China; ^5^National Clinical Research Center for Geriatric Disorders, Xiangya Hospital, Central South University, Changsha, China; ^6^The Charles B. Stout Neuroscience Mass Spectrometry Laboratory, Department of Neurology, College of Medicine, University of Tennessee Health Science Center, Memphis, TN, United States

**Keywords:** pituitary adenoma, signaling pathway, molecular network, multi-omics, personalized medicine, precision medicine, therapeutic target, molecule-panel biomarker

Pituitary adenoma is a common intracranial tumor that occurs in the pituitary gland that seriously affects the hypothalamus–pituitary–targeted organ axis system (1–3), and it is also a chronic, complex, and whole-body disease, with multiple causing factors, multiple processes, and multiple consequences (4, 5). It is very difficult to use any single molecule as biomarker to clarify its molecular mechanisms, and to predict, prevent, diagnose, and treat a pituitary adenoma (5–9). Rapidly developed multi-omics and systems biology affect treatment of pituitary adenomas and change the paradigms from the traditional single-factor strategy to a multi-parameter systematic strategy (5, 9–11). A series of molecular alterations at different levels of genes (genome), RNAs (transcriptome), proteins (proteome), peptides (peptidome), metabolites (metabolome), and imaging characteristics (radiome) that resulted from exogenous and endogenous carcinogens are involved in pituitary tumorigenesis, and mutually associate and function in a molecular network system (4, 5, 10, 11). Molecular network alterations are the hallmark of, and play central roles in, pituitary pathogenesis (12, 13). A key molecule-panel biomarker that is derived from a molecular network is necessary for accurate clinical practice of a pituitary adenoma (13). The modern multi-omics, computation biology, and systems biology technologies lead to the possibility of recognizing really reliable molecular-panel biomarkers for research and clinical practice in pituitary adenomas.

This present issue mainly focuses on the molecular network study of pituitary adenomas, which contains 14 topics: (i) The first topic addressed the idea that a tyrosine kinase inhibitor, imatinib, inhibited growth hormone (GH) secretion signaling via the PDGFR-β pathway, but did not affect cell viability and apoptosis, which might be used as an adjunct therapy to treat GH-secreting pituitary adenomas (Gupta et al.). These data clearly demonstrate the importance of GH signaling pathway-based therapeutic treatment in GH-secreting pituitary adenomas. (ii) The second topic addressed the notion that the pattern change of six prolactin (PRL) proteoforms existed among five subtypes of pituitary adenomas, and different hPRL proteoforms might function in different PRL receptor-signaling pathways, which clearly demonstrates the importance and clinical value of PRL receptor-signaling pathway-based PRL proteoform pattern study in human pituitaries and pituitary adenomas (Qian, Yang et al.). (iii) The third topic addressed the integration of proteomics and metabolomics to reveal metabolite–protein networks in adrenocorticotropic hormone (ACTH)-secreting pituitary adenomas (Feng et al.). Proteomic and metabolomic variations are a precious resource to clarify disease mechanisms and determine effective biomarkers (14). This topic emphasized the importance of the protein–metabolite joint pathway analysis and revealed glycolysis/gluconeogenesis, pyruvate metabolism, citrate cycle (TCA cycle), fatty acid metabolism, and Myc signaling pathways in ACTH-secreting pituitary adenomas. (iv) The fourth topic addressed estrogen signaling pathway-mediated sex difference in lactotroph tumor aggressiveness and discovered a number of estrogen receptor (ER)-related candidate genes as target molecules for sex-specific aggressive behavior in male lactotroph tumors (Wierinckx et al.). (v) The fifth topic addressed metabolomics as a promising approach to pituitary adenomas (Pînzariu et al.). Pituitary adenoma is an endocrine- and metabolic-related disease. Some metabolites have been studied; however, real metabolomics has not been extensively carried out in pituitary adenomas. (vi) The sixth topic addressed the new progress on different pathway mechanisms of dopamine and dopamine agonists (BRC and CAB) in prolactinomas (Liu et al.), which will provide new evidence for dopamine signaling pathway-based personalized and precise treatment of prolactinomas. (vii) The seventh topic addressed the molecular network basis of invasive pituitary adenomas, namely, the invasiveness-related molecules, including pituitary tumor transforming gene (PTTG), vascular endothelial growth factor (VEGF), hypoxia-inducible factor-1a (HIF-1a), fibroblast growth factor-2 (FGF-2), and matrix metalloproteinases (MMPs, mainly MMP-2, and MMP-9), which interact in a complex molecular network and are responsible for the invasiveness of pituitary adenomas (Yang and Li). Of course, the mechanism of the invasiveness of pituitary adenomas is very complex. Multiomics-based molecular network investigation might provide novel insights into the molecular mechanisms and therapeutic targets of pituitary adenoma invasiveness (5, 11). (viii) The eighth topic addressed the roles of phosphodiesterases (PDEs) and cAMP pathway in pituitary adenomas, which emphasizes that the unique disturbance of the cAMP-PDE pathway in most AIP-mutation positive pituitary adenomas could contribute to poor response to somatostatin analogs for personalized treatment of pituitary adenomas (Bizzi et al.). (ix) The ninth topic addressed the epigenomics of pituitary adenomas, focusing on DNA methylation, histone modification, and transcript modification (Hauser et al.). In-depth investigation of the relationship between tumor epigenetics-related molecular pathways and clinical pathological characteristics might serve for clinical decision-making. (x) The 10th topic addressed the first ubiquitinomic profile and ubiquitination-involved signaling pathway network alterations, including ribosome, hippo signaling pathway, PI3K-AKT signaling pathway, and nucleotide excision repair pathway, in human pituitary adenomas (Qian, Zhan et al.). (xi) The 11th topic addressed mitogen-activated protein kinases (MAPKs; including ERK, p38, and JNK) pathway-based drug therapeutic targets in pituitary adenomas (Lu et al.), which in detail discussed the advances in understanding the role of MAPK signaling in pituitary tumorigenesis, and the MAPK pathway-based potential therapeutic drugs for pituitary adenomas. (xii) The 12th topic addressed biological roles and mechanisms of mitochondrial dysfunction pathway network and mitochondrial dynamics in pituitary adenomas, and current status of mitochondria-based biomarkers and targeted drugs for effective treatment of pituitary adenomas (Li and Zhan). (xiii) The 13th topic described the large-scale quantitative proteomic profile (*n* = 6,076 proteins) and the protein molecular pathway network profile of non-functional pituitary adenomas, which were combined with transcriptomic data (*n* = 3,598 differentially expressed genes) to reveal 52 statistically significant pathways, including cGMP-PKG pathway, focal adhesion, and platelet activation signaling pathways (Cheng et al.). (xiv) The 14th topic addressed multiomics-based signaling pathway network alterations in non-functional pituitary adenomas (Long et al.), which analyzed nine sets of omics data, and provided a comprehensive and large-scale pathway network data for non-functional pituitary adenomas to understand the accurate molecular mechanisms and discover effective biomarkers for diagnosis, prognosis, and determination of therapeutic targets for pituitary adenomas.

Molecular networks are an effective approach to annotate the interactome in pituitary adenomas for in-depth insight into its molecular mechanisms and discovery of effective biomarkers and therapeutic targets (12, 13). (i) This special issue covers several single signaling pathways and their targeted therapeutic drugs. These single signaling pathways include the MAPK (ERK, p38, and JNK) signaling pathway (Lu et al.), mitochondrial dysfunctional pathway (Li and Zhan), GH-PDGFR-β signaling pathway (Gupta et al.), estrogen signaling pathway (Wierinckx et al.), cAMP-PDE pathway (Bizzi et al.), and dopamine signaling pathway (Liu et al.). Based on these given signaling pathways, some therapeutic targets and drugs have been discovered and FDA-approved for pituitary adenomas. However, one must realize that although many advances have been made, these signaling pathways have not been fully clarified in pituitary adenomas, and in-depth exploring diversity of each of these pathways might discover great potentials of these pathways for pituitary adenomas. Also, from a systematic viewpoint, the multi-target combination treatment within each pathway or with combining each pathway with other pathways will be superior to the single-target treatment. (ii) Multiomics has driven molecular network study in pituitary adenoma. This special issue involved in egigenomics (Hauser et al.), transcriptomics, proteomics (Cheng et al.), ubiquitinomics (Qian, Zhan et al.), nitroproteomics, phosphoproteomics, metabolomics (Pînzariu et al.; Feng et al.), and multiomics-based integrative study (Long et al.) in pituitary adenomas, including GH-secreting adenoma, ACTH-secreting adenoma, PRL-secreting adenoma, and non-functional pituitary adenomas. Omics-based molecular network analysis has made significant advances in pituitary adenomas. Until now, transcriptomics and proteomics have been extensively studied in pituitary adenomas (Cheng et al.) (15–19); epigenomics, ubiquitinomics, phosphoproteomics, nitroproteomics (20–23), and metabolomics have been initiated but not extensively studied in pituitary adenomas. However, one must note that post-transcriptional modifications/post-translational modifications (PTMs) are very complex up to several hundreds of PTMs in the human body (14), and PTM-mediated molecular network alterations play important roles in pituitary adenomas. However, PTM-omics has not been extensively studied in pituitary adenomas. Therefore, we would emphasize the scientific importance of PTM-omics, including DNA modifications, RNA modifications, and protein modifications, in pituitary adenomas. PTM-based omics and molecular network studies will bring the big promise for insight into the novel molecular mechanism, discovery of novel effective therapeutic targets and drugs, and determination of effective and reliable biomarkers for patient stratification, diagnosis, and prognostic assessment of pituitary adenoma patients. (iii) Pituitary adenoma invasiveness is a big clinical challenge. This special issue has one topic to address the molecular network basis of invasive pituitary adenoma based on several invasiveness-related molecules (PTTG, VEGF, HIF-1a, FGF-2, and MMPs such as MMP-2, and MMP-9) and their interacted complex molecular network (Yang and Li). However, one must note that these invasiveness-related molecules are derived from previous traditional studies and do not represent at all the entire molecule world of the invasive characteristics of pituitary adenomas. Indeed, the molecule world of invasive pituitary adenomas is very complex. We strongly recommend the use of multiomics to study pituitary adenoma invasiveness, which might be the right way to resolve its clinical invasiveness challenge for clarification of its molecular mechanisms, discovery of effective therapeutic targets, and determination of effective biomarkers for diagnosis and prognostic assessment. Some proteomics and transcriptomics between invasive and non-invasive pituitary adenomas have been performed to understand molecular mechanism and discover biomarkers of pituitary adenoma invasiveness (24–26). (iv) Proteome is the final functional performer of genome and transcriptome. However, proteome complexity is significantly influenced by RNA splicing, PTMs, and many other factors (14). The concept development of proteoform/protein species significantly enriches the content of proteome; a protein is an umbrella term of proteoform encoded by the same gene, and a proteoform is defined as its amino acid sequence + PTMs + spatial conformation + cofactors + binding partners + localization + a function, and thus proteoform is the basic unit of proteome (27, 28). Clarification of proteoforms and proteoform-mediated signaling pathway networks will precisely help understand the molecular mechanism, directly identify reliable biomarkers for precise diagnosis and prognostic assessment, and precisely determine therapeutic treatment of pituitary adenoma (28, 29). For pituitary adenoma, we have studied hormone proteoforms and their involved signaling pathway alterations (30), including GH proteoforms (31), and prolactin proteoforms (Qian, Yang et al.). Also, dopamine receptor proteoforms (Liu et al.), PDE proteoforms (Bizzi et al.), and their involved molecular signaling pathways are discussed in this special issue. Proteoform studies will need in-depth insights into a proteome, which is the future direction of proteomics. We recommend the strengthening of proteoform-mediated molecular signaling pathway network studies in pituitary adenomas for precise treatment in the future.

In summary, molecular network studies of pituitary adenoma have achieved significant advances. However, one must realize that this special issue contains only a fraction of the very important molecular network study of pituitary adenomas. This Research Topic serves as a spur to stimulate and encourage researchers who study molecular networks to come forward with its scientific merits to research and clinical practice of pituitary adenomas. Future issues will collect more multiomics-based molecular network studies with large-scale clinical information for basic research, translational research, and clinical practice in pituitary adenomas. We strongly believe that multiomics-based molecular network studies, molecular network-based therapeutic target and drug studies, and molecular network-based pattern biomarker studies (5, 10, 11, 13, 32–37) will bring a brighter future for pituitary adenoma patients through the realization of personalized and precision medicine.

## Author Contributions

XZ conceived the concept, designed manuscript, wrote and critically revised the manuscript, and was responsible for its financial supports and the corresponding works. DD participated in the development of concept and critically revised the manuscript. All authors approved the final manuscript.

### Conflict of Interest

The authors declare that the research was conducted in the absence of any commercial or financial relationships that could be construed as a potential conflict of interest.
